# Tenth Annual Meeting of the Mississippi Valley Association of Dental Surgeons

**Published:** 1854-04

**Authors:** A. M. Leslie


### *Tenth Annual Meeting of the Mississippi Valley Association of Dental Surgeons.*

Society met, according to adjournment, in the Ohio College of Dental Surgery, Wednesday morning, Feb. 22, 1854.

President, Dr. C. Bonsall, in the chair.

Present—Drs. Baxter, Goddard, Berry, Taft, Watt, Ulrey, Allen, Taylor.

The examining committee reported the names of the following gentlemen for membership in this society, and recommended their admission, viz.: Drs. H. R, Smith, Terre Haute; E. Collins, Connersville, Ia.; E. A. Herman, Nashville, Tenn., and M. DeCamp, Mansfield, O.

On motion, report accepted, and ballot had, when each was declared unanimously elected.

On motion, the chairman was requested to fill the executive committee, the members elect being absent.

The Chair appointed Drs. H. R. Smith, Taft, and Ulrey, on said committee.

It being called for, the President read the opening address; which, on motion of Dr. Taft, was requested for publication.

On motion, adjourned, to meet at 2 P.M.

*Afternoon Session.*—Present—Drs. Berry, Taft, Watt, Bonsall, Smith, Taylor, Leslie, Goddard, Ulrey, Baxter, and Allen, acting members.

On motion of Dr. Goddard, the following gentlemen, who were present, were invited to take seats during our sessions:

Drs. J. McClure, Carrolton, Miss.; Wm. A. Pease, Dayton, O.; W. J. Castner, Clarksville, Tenn.; W. R. Webster, Richmond, Ia.; B. T. Pleasants, Raymond, Miss.; H. Oliver, Zanesville, O.; Professor J. B. Smith, Cin., O; A. S. Dwyer, Louisville, Ky.; B. N. Freeman, Allegan, O; Jas. Bryson, Somerville, Tenn; J. H. Wood, Cin., O.; W. A. Cornelius, Covington, Ky.; Jos. Richardson, Cin., O.; J. B. Rottenstien, Galveston, Texas; A. L. Brown, Xenia, O.; J. N. Vaughn, Hopkinsville, Ky.; W. P. Price, Grenada, Miss.; Aaron S. Talbert, Lexington, Ky.; Geo. Evans, Lexington, Ky.; M. W. S. Kendall, Cin., O.; S. Wardle, Cin., O.; H. A. McDaniels, Cin., O.; Wm. M. Hunter, Cin., O.; E. G. Darling, Cin., O.; T. L. Hamlen, Cin., O.

President, Dr. Bonsall, in the chair.

Minutes read and approved.

Executive committee presented their report:

*Report of the Executive Committee.*—Your Executive Committee would respectfully submit the following report, in regard to the order of business:

1. Report of officers.
2. Report of standing committees.
3. Essays.
4. Miscellaneous business.

1st. Is the operation commonly denominated Risodontrypy, one really possessing the character claimed for it; and is it one warranted by the known laws of *Physiology* and *Pathology*.

2d. The best methods for making atmospheric pressure plates.

3d. The experience of the members in the use of Asbestus, or any other material, for filling the bottoms of cavities in tender teeth.

4th. Is the recent preparation of gold, known as sponge gold, crystal gold, &c., equal, or superior to foil, for filling teeth?

5th. Is the destruction of exposed nerves, preparatory to filling, advisable; if so, the best manner of performing the operation?

6th. The best metals for casts.

7th. Is block tin admissible for the construction of temporary sets of teeth? if so, what is the best method of working it?

8th. Election of officers be the special business for Thursday morning, at 11 o'clock.

H. R. Smith,

I. Taft, *Comm.*

J. P. Ulrey,

Which, on motion, was accepted.

Under the first item, the examining committee reported the following names, and recommended the gentlemen for membership in the society, viz.: Dr. John McClure, Carrolton, Miss., and Dr. Wm. A. Pease, Dayton, Ohio.

On motion, report was accepted, and ballot had; when they were declared unanimously elected.

The Treasurer presented his report, showing in his hands a balance of $186.26.

Drs. Goddard, Berry, and Baxter, were appointed to audit the same, and report.

Report from committee on Dental Progress being called for, Dr. Allen, the chairman stated, he had not prepared a report, having forgot that he had been appointed.

The committee appointed at the session of 1853 to receive and examine essays competing for the premiums offered at that meeting, reported that no essays had been received by them.

It being moved by Dr. Watt, that the resolutions of last year be re-adopted, on suggestion of Dr. Goddard, they were taken up separately; and, on his motion, the first was amended, so as to make the sum $100.

On motion of Dr. Leslie, it was further amended, so that the essay reported by the committee as the successful one, shall be printed and circulated among the members of the society, and shall not be issued for *general* circulation, until it shall receive the approval of the society; and, further, that the copyright become the property of the society.

The first resolution, as amended, was then adopted.

On motion, second resolution was amended, so as to make the committee five, and elect them by ballot.

As amended, the resolutions read:

1st. Resolved—That this association will award one hundred dollars, to the author of the best essay, (not previously published,) on Dental Surgery, *adapted to popular circulation.* To contain not less than fifty, nor more than seventy-five pages duodecimo; the copyright to be the property of the society. Said essay to be approved by a committee, and by it to be printed and issued to the members of the society, and receive the approval of the association before it be pub lished for general circulation.

2d. *Resolved,* That a committee of five be appointed, by ballot, to examine the essays presented, and report at next meeting. Essays competing for said award to be forwarded to the chairman of the committee as early as January 1st, 1855.

Election of said committee was had, and the following gentlemen appointed: Jas. Taylor, W. H. Goddard, A. Berry, A. M. Leslie, and J. Taft.

NOTE.—Authors sending essays under the above resolutions, will accompany it with their full signature and address, which they will inclose in a sealed envelope, which will be opened should the accompanying essay prove the successful one; otherwise, it will be returned with the manuscript.

The examining committee made a further report, presenting the names of Drs. A. S. Talbert, Lexington, Ky., Geo. W. Evans, Lexington, Ky., and W. R. Webster, Richmond, Ia., and recommended their admission into the association.

On motion, report accepted, and ballot gone into, when these gentlemen were declared duly elected.

The society proceeded to the consideration of the first item, under the head of Miscellaneous Business, in the report of the executive committee.

After the expression of the views of many members, the further discussion was laid over until Thursday morning.

The auditing committee presented the following:

Your auditing committee would respectfully report that they have examined the report of your Treasurer, and find, from his books and papers, that he has in his hands the following:

| | |
|---|---|
| Balance from last year - - - - | $125 26 |
| Received this session - - - - | 61 00 |
| | $186 26 |
| *Cr.* | |
| Paid Janitor - - - - - | 3 00 |
| Balance in hand - - - - | $183 26 |

W. H. GODDARD,

A. BERRY,

J. W. BAXTER, } *Committee.*

On motion, adjourned to 10½, A. M., to-morrow.

*Second day—Morning Session.*—The President in the chair.

Minutes of previous meeting read and approved.

The consideration of Risodontrypy was resumed, and the discussion continued; after which, on motion, a committee, consisting of Drs. Goddard, Taft, and Ulrey, was appointed, to examine forceps, presented under the premium resolution of last year.

The special order for 11 o'clock being the election of officers, the following gentlemen, after balloting, were declared elected:

President—W. H. Goddard, Louisville, Ky.

Vice-president—A. Berry, Covington, Ky.

Recording Secretary—A. M. Leslie, Cincinnati, O.

Corresponding Secretary—George Watt, Xenia, O.

Treasurer—C. Bonsall, Cincinnati, O.

Examining Committee—Drs. Jonathan Taft, Xenia, O.; H. R. Smith, Terre Haute, Ia.; J. P. Ulrey, Lawrenceburgh, Ia.

Executive Committee—Drs. W. R. Webster, Richmond, Ia.; A. S. Talbert, Lexington, Ky.; J. W. Baxter, Warsaw, Ky.

Committee on Dental Progress—Drs. A. M. Leslie, Cin., O.; George Watt, Xenia, O.; Wm. A. Pease, Dayton, O.

Dr. Goddard moved the adoption of the following resolution:

*Resolved,* That the members of the Miss. V. Association of Dental Surgeons, are extremely gratified, and hail with pleasure, the ensuing meeting of the American Society of Dental Surgeons, to be held in the city of Cincinnati in August next; and we pledge ourselves to use our best endeavors to greet the brethren of our sister association, and extend them a hearty welcome. We hope a large delegation will be present.

Dr. A. Berry moved the adoption of the following resolution:

*Resolved,* That the resolution passed by this society in 1844, in reference to mineral compounds for filling teeth, be hereby rescinded.

After discussion by some of the members, it was, on motion of Dr. Leslie, laid on the table, and made the special business at 3 o'clock.

The society took up the subject of atmospheric plates, as suggested by the executive committee.

On motion, the order of business was suspended, that the committee on forceps might report.

*Report of Committee on Forceps.*—Your committee have had presented to them a part of a set of extracting instruments, viz.: 18 pairs of forceps, of every variety of shape, crook, and adaptation. But, as the resolution of the society calls for a perfect set of extracting instruments, which your committee suppose, includes every instrument required by a dentist to remove teeth, fangs, etc. The set before us being incomplete, we deem it unnecessary to make any further report.

Your committtee, in justice to the cutler, Mr. H. R. Sherwood, of Cincinnati, would state, that his workmanship, (judging from the forceps before them,) is unsurpassed by any we have before seen, and would cheerfully recommend him to the favorable consideration of the Dental profession.

W. H. GODDARD,<br>J. TAFT,<br>J. P. ULREY, } *Committee.*

On motion, adjourned to 2 P.M.

*Afternoon Session.*—Society met, according to adjournment. President in the chair.

Minutes of morning session read and approved.

Dr. H. R. Smith moved that a committee of three be appointed, to report what may be considered as constituting a perfect set of extracting instruments. Lost.

On motion of Dr. Leslie, the resolution of last year, offering a premium on extracting instruments, was amended, so as to read:

*Resolved,* That this association will award fifty dollars, or a medal of equal value, on the most perfect set of extracting instruments, the same to receive the approval of the society.

It is understood this is open to all members of the profession, as well as to manufacturers.

Dr. Bonsall left the chair, and conducted Dr. Goddard, the president elect to the same; who, in a few brief remarks, tendered his thanks to the society, for the honor conferred in again calling him to preside over them.

On motion,

*Resolved,* That the thanks of this association be tendered to Dr. Bonsal for the faithful and impartial manner in which he has presided the past year.

Dr. Pease moved that the chair appoint a committee of three, to ascertain the average duration of clasp teeth and report at session of 1855.

The president appointed Drs. Pease, Watt, and McCullom, said committee.

Dr. Watt presented to the members of the society an improved screw for extracting stumps of teeth. The screw is separable from the shank, and sits in a square socket. After insertion, the handle is removed, and the forceps applied.

The resolution laid over from the morning was next discussed at length by many members; after which, on his request, the mover had leave to withdraw it

Moved, that the rules be suspended, and item 4th be next considered; carried.

On motion, adjourned, to mcet at 7 P. M.

*Evening Session.*—Vice-President, Dr. A. Berry, in the chair.

Minutes of afternoon session read and approved.

The subject of "sponge gold, crystal gold," etc., was further discussed by several members; explained at length by Dr. Taft, who spoke of some of the modes of preparing crystal gold; and pledged, that so soon as some experiments he and Dr. Watt are pursuing, are sufficiently progressed, they will describe the processes minutely, for the general benefit of the profession.

Under the rules, the 3d item of "miscellaneous business" was discussed; and the use of asbestos under the fillings, in cases of highly sensitive dentine, generally recommended.

On motion of Dr. Leslie,

*Resolved,* That a committee be appointed to procure the printing of five hundred copies of the constitution and by-laws, in pamphlet form, with power to draw on the treasurer for amount of bill. The chair appointed Drs. Leslie, Taylor, and Bonsal.

On motion of Dr. Taft,

*Resolved,* That the final adjournment to-morrow be not later than 12 M.

Dr. Pease read some tables, showing "the comparative periods of decay of the bicuspid and molar teeth, at different periods of life, in the sexes."

Adjourned to 8 A. M., to-morrow.

*Third day. Morning Session.*—Minutes read and approved.

On motion of Dr. Taft,

*Resolved,* That a committee be appointed to conduct microscopic observations, and to report the same to the next meeting.

Dr. Taylor moved the chair appoint a committee of three on the above.

Chairman appointed Drs. Taft, Taylor, and Leslie, said committee.

The 5th item of M. B. was discussed by several members, and the destruction of exposed nerves, preparatory to filling, generally approved—especially in incisors and bicuspides. For its destruction, an instrument is preferred when the nervous condition of the patient will allow it; otherwise, arsenious acid was considered the best chemical agent to be used in its destruction.

6th item, viz.: "Best metal for casts."

An alloy of two or more metals preferred by some members for male casts. Some use tin and zinc; others, copper and tin, &c.

7th item considered.—Dr. Baxter addressed the society on the subject, and presented a mode he has pursued, (it being a modification of Hawe's); and presented to the society a full set of gum teeth he had mounted on his plan.

On motion of Dr. H. R. Smith,

*Resolved,* The thanks of this society be tendered Dr. Baxter, for his specimens and explanation of the mode; and that he be requested to furnish a written description for publication.

On motion of Dr. Leslie,

*Resolved,* That the bye-laws be amended, by the insertion of the following, (taken from the resolutions on record,) as the 7th article: That any member of this society who shall extol his own peculiar merits over those of a fellow practitioner, or offer his services at lower rates than is common among the members of the profession among whom he operates, through the public prints, or use any secret nostrum, (unless pledged prior to the present time, 1846, to maintain secresy,) shall be liable to expulsion from the society.

Dr. Leslie moved the following be inserted as the eighth article:

"That any member of this society who may patent any instrument, or mode of practice, may be subject to expulsion from the society."

This was not passed. Members expressed their satisfaction with the resolution of the committee on patents which was adopted in 1852, and a request made that said resolution be printed with the constitution.

On motion of Dr. Leslie, the following was added to the sixth section of article 1st: The Treasurer shall also notify members in arrears two years; and, if not paid by the meeting next succeeding said notice, it shall be his duty to report the same to the society.

The original seventh article was adopted as the ninth.

The following gentlemen were appointed to prepare essays for next year:

Opening address—By the President.

Essays—A. S. Talbert, H. R. Smith, Geo. Watt, J. G. Hamill, J. P. Ulrey, and H. McCullom.

The secretary drew the attention of the society to the fact that it possessed no place suitable for the preservation of the papers, books, specimens, etc., it may come into the possession of, and which it would no doubt receive if the proper means were adopted to form a cabinet. This he thought a favorable opportunity, (the college association being about to rebuild,) to secure suitable accommodations.

On motion of Dr. Taft,

*Resolved,* That a committee of three be appointed to confer with the building committee of the College Association, with power to make such arrangements as may seem best, and draw on the treasurer for the necessary funds.

The chair appointed Drs. Leslie, Bonsall, and Taylor, said committee.

On motion, the treasurer was authorized to pay the janitor of O. D. College, $5 for services.

Minutes read and approved.

On motion, adjourned, to meet in the Ohio College of Dental Surgery, Wednesday, 10 A. M., February 23, 1855.

A. M. Leslie, Secretary.

---

Dr. Chas. Bonsall's *Address before the Missisippi Valley Association of Dental Surgeons.*

